# Respiratory syncytial virus is an “opportunistic” killer

**DOI:** 10.1002/ppul.23963

**Published:** 2018-02-20

**Authors:** Mauricio T. Caballero, Fernando P. Polack

**Affiliations:** ^1^ Fundacion INFANT Buenos Aires Argentina; ^2^ Department of Pediatrics Vanderbilt University Nashville Tennessee

**Keywords:** congenital heart disease, mortality, pneumonia, pneumothorax, respiratory syncytial virus

## Abstract

Respiratory syncytial virus (RSV), responsible for more than three million yearly hospitalizations and up to 118 000 deaths in children under 5 years, is the leading pulmonary cause of death for this age group that lacks a licensed vaccine. Ninety‐nine percent of deaths due to the virus occur in developing countries. In‐hospital RSV fatalities affect previously healthy term infants in association with bacterial sepsis, clinically significant pneumothoraxes and, to a lesser extent, comorbid conditions. Community deaths affect low‐income children from socially vulnerable families and appear to be as frequent as inpatient fatalities. In industrialized countries, RSV deaths occur almost exclusively in children with premorbid conditions.

In a sense, RSV is an “opportunistic” killer. It needs a synergistic premorbid, medical practice‐related, infectious, or social co‐factor to cause a fatal outcome. But while the complex problems associated with these co‐factors await solutions, candidate vaccines, long‐lived monoclonal antibodies and antivirals against RSV are under clinical evaluation. It seems reasonable to predict that the landscape of RSV infections will look different in the next decade.

## 1 RESPIRATORY DISEASES ARE A LEADING CAUSE OF MORTALITY IN YOUNG CHILDREN

The burden of child mortality is overwhelmingly inflicted on low‐income countries.^1^ There, children die in excess of 20‐fold more than in industrialized nations.^2^ Neonatal deaths (0‐28 days) are frequent, particularly early after birth.^3^, ^4^ And the proportion of deaths in the post‐neonatal period (29‐364 days) is higher than in industrialized countries.^3^ Pneumonia and diarrhea are responsible for most fatalities during post‐neonatal infancy and early childhood.^5^

In recent decades, conjugate vaccines have been licensed against the main causes of death associated with pneumonia, *Streptococcus pneumoniae* and *Haemophilus influenzae* type B.^6^ As a consequence, the leading pulmonary cause of death in this age group for which no vaccine is available is respiratory syncytial virus (RSV).^7^ A recent systematic review of 329 published and unpublished studies estimated that RSV was responsible for 3.2 (2.7‐3.8) million hospitalizations and up to 118 000 deaths in children under 5 years of age during 2015.^8^

## 2 CHALLENGES IN ESTIMATING RSV MORTALITY RATES

Assessing the burden of RSV mortality is challenging.^9^ First, most deaths caused by RSV in developing countries occur in regions with limited access to viral testing. In fact, even when testing is available, physicians do not prioritize obtaining a nasal swab or nasopharyngeal aspirate in critically ill patients to detect a viral illness with no specific treatment.^10^ Therefore, unless a study is designed to ascertain the role of RSV in life threatening and fatal infections, its burden will be significantly underestimated. Second, hospitals in low‐income regions assay respiratory samples for RSV using rapid detection techniques, most often direct immunofluorescence assays.^8^ These assays rely heavily on the individual expertise of the laboratory staff, and consequently vary in sensitivity and specificity.^8^ In 2011, using RT‐PCR as gold standard, we surveyed hospital immunofluorescence reports for RSV in twelve hospitals of a low‐income region in Argentina and found sites with sensitivities as low as 22% and others with high rates of false positives (FPP, unpublished information). Third, using flu criteria as a surrogate for diagnosis of RSV in infants is hampered by the absence of fever in half of RSV cases.^11^ Moreover, attributing all fatalities with a clinical diagnosis of bronchiolitis to RSV is also troublesome. Symptoms of bronchiolitis can be elicited by a variety of viruses that co‐exist during the respiratory season and their individual case fatality ratios (CFR) remain unclear.^8^, ^10^, ^12^, ^13^ RSV is a more frequent cause of severe LRTI in children than other respiratory pathogens, often detected in 50‐70% of hospitalizations, but it also may be less lethal.^10^, ^13^, ^14^ In addition, fatalities associated with RSV often follow the virus' seasonal peak by weeks, as secondary bacterial infections appear to play an important role in the process.^13^, ^15^, ^16^ In fact, peak pneumococcal mortality is closely linked to and can temporally follow RSV activity.^16^ Therefore, focusing on viral symptoms to define mortality rates may be misleading. Fourth, infants can die at home from or with an undetected RSV LRTI.^9^ Home deaths occur in clinically frail infants with sick lungs or simply in those exhausted due to lack of medical care or oxygen supplementation. In many developing countries, the number of home deaths during the respiratory season significantly exceeds the number of hospital deaths.^13^ Finally, pathogens detected in the upper respiratory tract of deceased infants may not necessarily match those identified in the lungs, where more than one pathogen is routinely detected by molecular techniques during autopsies.^9^ Hence, cause of death attribution is complex and often dependent on criteria pre‐defined by investigators.

## 3 MORTALITY DUE TO RSV IN INDUSTRIALIZED COUNTRIES

Deaths attributable to RSV in industrialized countries are infrequent and occur in children with premorbid conditions. Case fatality ratio meta‐estimates for 2015 in infants 0‐5 and 6‐11 months of age were 0.2 (0.0‐12.8) and 0.9 (0.2‐4.0), respectively, in high income countries.^8^ A U.S. study queried large administrative databases using data coded from inpatient encounters to update decades‐old mortality estimates and explore conditions associated with fatal outcomes.^17^ Deaths were infrequent, occurring in 3‐4/10 000 admissions in those with a primary diagnosis of RSV. The mean annual national mortality estimate due to RSV was 56‐121 deaths at a mean age of 6.2‐7.5 months. Fatalities associated with complex chronic conditions in 76‐79% of cases. The most frequent comorbidities were cardiovascular (37‐45%), neuromuscular (20‐26%), respiratory (19‐21%), and genetic or congenital (13‐19%). More than one condition was present in 37‐39% of patients.^17^

Recently, a retrospective study recovered risk factors for mortality in 358 children from 23 countries across the world (68% from upper middle income and high‐income countries) and confirmed the influence of comorbid conditions in fatalities from industrialized nations (70% vs 28% reported in low or lower‐middle income countries).^18^ The most frequent comorbid condition was congenital heart disease (CHD). The mean age of death was slightly younger in the developing world (4 vs 7 months in industrialized countries). Mean time between onset of symptoms and admission ranged from 5 days in low income to 3 days in higher‐middle and high‐income countries, but less than 50% of families contacted a physician before hospitalization.^18^

Other children suffering chronic diseases that can experience severe RSV infections are transplant patients.^19^, ^20^, ^21^ While RSV‐attributable CFR are typically <10% in immunocompromised children, these numbers are higher in transplant patients. RSV CFR reported in children experiencing bone marrow transplants due to hematological malignancies and solid organ recipients are as high as 19% and 40%, respectively.^19^, ^20^, ^21^

## 4 IN‐HOSPITAL MORTALITY DUE TO RSV IN DEVELOPING COUNTRIES

The disparities in RSV mortality rates between countries and regions of different socioeconomic status are striking. CFR meta‐estimates for 2015 in infants 0‐5 and 6‐11 months of age in developing countries were 2.2 (1.8‐2.7) and 2.4 (1.9‐3.2), respectively.^8^ A second meta‐analysis identified the highest case fatality rates in Kenya, Morocco, South Africa, and Mozambique.^22^

RSV mortality in the developing world underscores the serious structural problems in public health services that govern the fate of many children worldwide.^8^, ^13^ After all, why would otherwise an ailment not treated by specific medication kill children so asymmetrically? Answers highlight a critical problem often faced by families living in poverty: the limited specific expertise of those caring for acute medical emergencies in infants and young children^13^.

In contrast to data from industrialized countries, a recent study in socially vulnerable infants in Argentina showed RSV LRTI to be the leading cause of post‐neonatal infant mortality.^13^ The CFR for infants due to RSV was 0.90% (95%CI, 0.44‐1.35) compared to 1.49% (95%CI 0.51‐2.47) for non‐RSV LRTI. However, because of its high hospitalization rates, RSV was responsible for 50% neonatal and 54% post‐neonatal infant deaths presenting with LRTI.

RSV deaths primarily occurred in previously healthy term infants in association with bacterial sepsis and clinically significant pneumothoraxes. The frequency of pneumothorax in hospitalized infants with fatal RSV LRTI (40%) grossly outnumbered rates in intensive care units in industrialized countries.^13^, ^23^ Mechanical ventilation in RSV‐infected patients with segmental atelectasis and lung hyperinflation may require high volumes to overcome hypercapnia, increasing the risk of air leaks.^24^ As infants from low‐income countries progressively access these potentially lifesaving technologies, expert training of health care personnel will be critical to prevent excess mortality.

In addition, *Staphylococcus aureus* and *S. pneumoniae* sepsis played a pivotal role in RSV‐related mortality. In fact, a study of lung sections from children younger than age 2 years who died with pneumonia in Mexico detected RSV in 30% of 98 subjects between 1989 and 1997.^25^ RSV causes functional changes in respiratory epithelial cells facilitating adherence and invasion of bacteria.^26^ Therefore, higher nasopharyngeal carriage rates of bacteria in infants from developing countries may contribute to excess invasive disease.^16^ Only 10% RSV deaths in the Argentinean study did not have sepsis and/or a pneumothorax.^13^ The role of age and typical risk factors for severe LRTI was less ostensible, but CHD, neurological illness, and Down's syndrome associated with poor outcomes.^13^

## 5 COMMUNITY DEATHS DUE TO RSV IN DEVELOPING COUNTRIES

Every year, many children in the developing world die at home from RSV LRTI.^8^, ^9^, ^13^ Worldwide estimates are difficult due to the scarcity of data, but a recent systematic review calculated home deaths at 49.6% of 118 200 RSV‐attributable fatalities in 2015.^8^ In Buenos Aires, Argentina, and Lombok, Indonesia pediatric community deaths were surveyed and shown to peak during the RSV season.^13^, ^27^ In addition to the seasonal overlap, age distribution for infant community deaths‐reported for Buenos Aires‐ paralleled that observed in infants hospitalized due to RSV LRTI.^13^

However, co‐factors contributing to home death in children infected with RSV may differ significantly from those observed at the hospital.^13^, ^28^ Some cases may affect children with pre‐existent lung ailments. For instance, evidence of chronic bronchitis due to regular exposure to airborne pollutants and smoke appear to be important in our population. Other infants appear to die “silent deaths,” exhausted from battling indolent respiratory distress due to lack of medical care and/or oxygen supplementation in overcrowded homes.

## 6 CONCLUSIONS

In summary, RSV is an “opportunistic” killer. It needs a synergistic co‐morbid,^17^, ^18^ medical practice‐related,^13^ infectious,^13^, ^16^ or social^8^, ^9^ factor to cause a fatal outcome. Infrequent deaths in industrialized countries associate with complex comorbidities ranging from chronic lung and heart disease to transplants.^17^, ^19^ In developing country hospitals, suboptimal medical care during emergencies and secondary bacterial infections can lead to poor outcomes.^13^, ^16^, ^25^, ^27^ And hazardous environmental exposures and social handicaps may team up to weaken the lung and/or delay medical care, converting a routine viral infection at home into a lethal ailment.

## 7 THE FUTURE

These are exciting times to participate in the global effort against RSV. A better understanding of viral structure and protective epitopes is sophisticating approaches to prevention against the pathogen.^29^, ^30^, ^31^ Several candidate vaccines for maternal or infant immunization and passive protection strategies for neonates and infants are undergoing clinical evaluation.^32^ Novel antiviral strategies are being studied in hospitalized patients.^33^ Numerous multicenter collaborations advance to clarify the role of the pathogen in long term morbidity (ie,: asthma) and infant mortality.^8^, ^9^, ^34^, ^35^ Recent reports are describing the consequences of infection in pregnant women.^3^, ^36^ Recently, Shi et al^8^ estimated that a successful maternal or newborn immunization strategy, with 80% protection and near universal coverage, could prevent 22 000 RSV‐associated fatalities a year.

However, challenges remain to improve intensive care practices and identify additional strategies to decrease the burden of secondary bacterial infections. Also, a better estimate of the true burden of RSV in community deaths is necessary. Recent efforts to incorporate a novel strategy, minimally invasive sampling of tissue (MITS) for histopathology, immunohistochemistry, and molecular studies, coupled with verbal autopsies and PCR detection of the virus in nasopharyngeal secretions should improve our understanding of the problem.^9^ In addition, respiratory virus surveillance will be critical after licensure of vaccines or antibodies to gauge their impact and monitor potential viral replacement, if RSV is cornered by these products.^37^ The emergence of antiviral drugs against respiratory pathogens in coming years may further encourage testing.^38^, ^39^

We are witnessing rapid advances in our understanding of RSV and its consequences. It seems reasonable to predict that the landscape of severe respiratory infections will look different in the next decade.

## References

[1] Lozano R , Naghavi M , Foreman K , et al. Global and regional mortality from 235 causes of death for 20 age groups in 1990 and 2010: a systematic analysis for the Global Burden of Disease Study 2010. Lancet. 2012; 380:2095–2128. 23245604 10.1016/S0140-6736(12)61728-0 PMC10790329

[2] The state of the world children 2008: child survival. https://www.unicef.org/sowc08/docs/sowc08.pdf. 2008. Accessed October 10th 2017.

[3] Wheeler SM , Dotters‐Katz S , Heine RP , Grotegut CA , Swamy GK . Maternal effects of respiratory syncytial virus infection during pregnancy. Emerg Infect Dis. 2015; 21:1951–1955. 26485575 10.3201/eid2111.150497 PMC4622246

[4] Bryce J , Boschi‐Pinto C , Shibuya K , Black RE . Group WHOCHER. WHO estimates of the causes of death in children. Lancet. 2005; 365:1147–1152. 15794969 10.1016/S0140-6736(05)71877-8

[5] Liu L , Oza S , Hogan D , et al. Global, regional, and national causes of under‐5 mortality in 2000–15: an updated systematic analysis with implications for the Sustainable Development Goals. Lancet. 2016; 388:3027–3035. 27839855 10.1016/S0140-6736(16)31593-8 PMC5161777

[6] Theodoratou E , Johnson S , Jhass A , et al. The effect of *Haemophilus influenzae* type b and pneumococcal conjugate vaccines on childhood pneumonia incidence, severe morbidity and mortality. Int J Epidemiol. 2010; 39:i172–i185. 20348119 10.1093/ije/dyq033 PMC2845872

[7] Kovacs SD , Mullholland K , Bosch J , et al. Deconstructing the differences: a comparison of GBD 2010 and CHERG's approach to estimating the mortality burden of diarrhea, pneumonia, and their etiologies. BMC Infect Dis. 2015; 15:16. 25592774 10.1186/s12879-014-0728-4 PMC4305232

[8] Shi T , McAllister DA , O'Brien KL , et al. Global, regional, and national disease burden estimates of acute lower respiratory infections due to respiratory syncytial virus in young children in 2015: a systematic review and modelling study. Lancet. 2017; 390:946–958. 28689664 10.1016/S0140-6736(17)30938-8 PMC5592248

[9] Zanone SM , Krause LK , Madhi SA , et al. Challenges in estimating RSV‐associated mortality rates. Lancet Respir Med. 2016; 4:345–347. 27304556 10.1016/S2213-2600(16)30042-X

[10] Berkley JA , Munywoki P , Ngama M , et al. Viral etiology of severe pneumonia among Kenyan infants and children. Jama. 2010; 303:2051–2057. 20501927 10.1001/jama.2010.675 PMC2968755

[11] Saha S , Pandey BG , Choudekar A , et al. Evaluation of case definitions for estimation of respiratory syncytial virus associated hospitalizations among children in a rural community of northern India. J Glob Health. 2015; 5:010419. 26649172 10.7189/jogh.05.020419 PMC4652925

[12] Ferolla FM , Hijano DR , Acosta PL , et al. Macronutrients during pregnancy and life‐threatening respiratory syncytial virus infections in children. Am J Respir Crit Care Med. 2013; 187:983–990. 23449690 10.1164/rccm.201301-0016OC

[13] Geoghegan S , Erviti A , Caballero MT , et al. Mortality due to Respiratory Syncytial Virus. Burden and Risk Factors. Am J Respir Crit Care Med. 2017; 195:96–103. 27331632 10.1164/rccm.201603-0658OC

[14] Cohen C , Walaza S , Treurnicht FK , et al. In‐ and out‐of‐hospital mortality associated with seasonal and pandemic influenza and respiratory syncytial virus in south Africa, 2009–2013. Clin Infect Dis. 2018; 66:95–103. 29040527 10.1093/cid/cix740 PMC5813484

[15] Stockman LJ , Brooks WA , Streatfield PK , et al. Challenges to evaluating respiratory syncytial virus mortality in Bangladesh, 2004–2008. PLoS ONE. 2013; 8:e53857. 23365643 10.1371/journal.pone.0053857 PMC3554708

[16] Wolter N , Tempia S , Cohen C , et al. High nasopharyngeal pneumococcal density, increased by viral coinfection, is associated with invasive pneumococcal pneumonia. J Infect Dis. 2014; 210:1649–1657. 24907383 10.1093/infdis/jiu326

[17] Byington CL , Wilkes J , Korgenski K , Sheng X . Respiratory syncytial virus‐associated mortality in hospitalized infants and young children. Pediatrics. 2015; 135:e24–e31. 25489019 10.1542/peds.2014-2151 PMC4279071

[18] Scheltema NM , Gentile A , Lucion F , et al. Global respiratory syncytial virus‐associated mortality in young children (RSV GOLD): a retrospective case series. Lancet Glob Health. 2017; 5:e984–e991. 28911764 10.1016/S2214-109X(17)30344-3 PMC5599304

[19] Manzoni P , Figueras‐Aloy J , Simoes EAF , et al. Defining the incidence and associated morbidity and mortality of severe respiratory syncytial virus infection among children with chronic diseases. Infect Dis Ther. 2017; 6:383–411. 28653300 10.1007/s40121-017-0160-3 PMC5595774

[20] Shah DP , Ghantoji SS , Ariza‐Heredia EJ , et al. Immunodeficiency scoring index to predict poor outcomes in hematopoietic cell transplant recipients with RSV infections. Blood. 2014; 123:3263–3268. 24700783 10.1182/blood-2013-12-541359 PMC4046424

[21] Tran TT , Gonzalez IA , Tekin A , McLaughlin GE . Lower respiratory tract viral infections in pediatric abdominal organ transplant recipients: a single hospital inpatient cohort study. Pediatr Transplant. 2013; 17:461–465. 23672407 10.1111/petr.12093 PMC7159153

[22] Stein RT , Bont LJ , Zar H , et al. Respiratory syncytial virus hospitalization and mortality: systematic review and meta‐analysis. Pediatr Pulmonol. 2017; 52:556–569. 27740723 10.1002/ppul.23570 PMC5396299

[23] Hennus MP , Janssen R , Pennings JL , et al. Host response to mechanical ventilation for viral respiratory tract infection. Eur Respir J. 2012; 40:1508–1515. 22496321 10.1183/09031936.00177111

[24] Given K , Schultz A , Douglas TA , Martin AC . Air leaks in children with acute bronchiolitis. J Paediatr Child Health. 2008; 44:604–606. 19012637 10.1111/j.1440-1754.2008.01393.x

[25] Bustamante‐Calvillo ME , Velazquez FR , Cabrera‐Munoz L , et al. Molecular detection of respiratory syncytial virus in postmortem lung tissue samples from Mexican children deceased with pneumonia. Pediatr Infect Dis J. 2001; 20:495–501. 11368106 10.1097/00006454-200105000-00005

[26] Hament JM , Aerts PC , Fleer A , et al. Enhanced adherence of Streptococcus pneumoniae to human epithelial cells infected with respiratory syncytial virus. Pediatr Res. 2004; 55:972–978. 15103015 10.1203/01.PDR.0000127431.11750.D9

[27] Nair H , Nokes DJ , Gessner BD , et al. Global burden of acute lower respiratory infections due to respiratory syncytial virus in young children: a systematic review and meta‐analysis. Lancet. 2010; 375:1545–1555. 20399493 10.1016/S0140-6736(10)60206-1 PMC2864404

[28] Shi T , Balsells E , Wastnedge E , et al. Risk factors for respiratory syncytial virus associated with acute lower respiratory infection in children under five years: systematic review and meta‐analysis. J Glob Health. 2015; 5:020416. 26682048 10.7189/jogh.05.020416 PMC4676580

[29] Palomo C , Mas V , Vazquez M , et al. Polyclonal and monoclonal antibodies specific for the six‐helix bundle of the human respiratory syncytial virus fusion glycoprotein as probes of the protein post‐fusion conformation. Virology. 2014; 460‐461:119–127. 10.1016/j.virol.2014.05.001 25010277

[30] McLellan JS , Chen M , Joyce MG , et al. Structure‐based design of a fusion glycoprotein vaccine for respiratory syncytial virus. Science. 2013; 342:592–598. 24179220 10.1126/science.1243283 PMC4461862

[31] McLellan JS , Chen M , Leung S , et al. Structure of RSV fusion glycoprotein trimer bound to a prefusion‐specific neutralizing antibody. Science. 2013; 340:1113–1117. 23618766 10.1126/science.1234914 PMC4459498

[32] PATH. RSV vaccine and mAb snapshot http://www.path.org/vaccineresources/details.php?i=1562. Accessed on 31st Ocober 2017.

[33] Mazur NI , Martinon‐Torres F , Baraldi E , et al. Lower respiratory tract infection caused by respiratory syncytial virus: current management and new therapeutics. Lancet Respir Med. 2015; 3:888–900. 26411809 10.1016/S2213-2600(15)00255-6

[34] Blanken MO , Rovers MM , Molenaar JM , et al. Respiratory syncytial virus and recurrent wheeze in healthy preterm infants. N Engl J Med. 2013; 368:1791–1799. 23656644 10.1056/NEJMoa1211917

[35] Caballero MT , Jones MH , Karron RA , et al. The Impact of Respiratory Syncytial Virus Disease Prevention on Pediatric Asthma. Pediatr Infect Dis J. 2016; 35:820–822. 10.1097/INF.0000000000001167 27351360

[36] Chu HY , Katz J , Tielsch J , et al. Clinical presentation and birth outcomes associated with respiratory syncytial virus infection in pregnancy. PLoS ONE. 2016; 11:e0152015. 27031702 10.1371/journal.pone.0152015 PMC4816499

[37] Achten NB , Wu P , Bont L , et al. Interference between respiratory syncytial virus and human rhinovirus infection in infancy. J Infect Dis. 2017; 215:1102–1106. 28368456 10.1093/infdis/jix031 PMC5426371

[38] DeVincenzo JP , Whitley RJ , Mackman RL , et al. Oral GS‐5806 activity in a respiratory syncytial virus challenge study. N Engl J Med. 2014; 371:711–722. 25140957 10.1056/NEJMoa1401184

[39] DeVincenzo JP , McClure MW , Symons JA , et al. Activity of oral ALS‐008176 in a respiratory syncytial virus challenge study. N Engl J Med. 2015; 373:2048–2058. 26580997 10.1056/NEJMoa1413275

